# Oral encapsulated vascular malformation: An undescribed presentation in the mouth

**DOI:** 10.4317/jced.52698

**Published:** 2016-02-01

**Authors:** Wilfredo-Alejandro González-Arriagada, Márcio-Américo Dias, Pedro-de Souza Dias, Marisol Martínez-Martínez, Marcondes Sena-Filho, Oslei-Paes de Almeida

**Affiliations:** 1 DDS, MSc, PhD. Oral Pathology and Diagnosis, Facultad de Odontología, Universidad de Valparaíso, Valparaíso, Chile; 2 DDS. Stomatology, Faculdade de Odontologia do INAPOS, Pouso Alegre, Minas Gerais, Brazil; 3 DDS. Faculdade de Odontologia da UNIFENAS, Alfenas, Minas Gerais, Brazil; 4 DDS, MSc, PhD. Oral Diagnosis Department, Piracicaba Dental School, State University of Campinas (UNICAMP), Piracicaba, Sao Paulo, Brazil

## Abstract

Vascular lesions have been classified in two broad categories, hemangiomas and malformations. Encapsulated vascular lesions have not been reported in the oral cavity, but they were described in other sites, mainly in the orbit. Herein, we present a case of an oral encapsulated vascular lesion located in the right buccal mucosa of a 69-year-old male, including histological and immunohistochemical description and a literature review.

** Key words:**Buccal mucosa, hemangioma, vascular malformation, oral cavity.

## Introduction

Classification of benign vascular lesions/tumors is complex and confusing, and they have been grouped in two broad categories, hemangiomas and vascular malformations (1,2). Hemangiomas are tumors of infancy and childhood, usually appearing few weeks after birth, and characterized by initial rapid growth during the first year of life, with subsequent slow involution over years (1,3,4). Vascular malformations are defined as congenital lesions that become apparent later in life, formed by dysplastic vascular channels, with no endothelial proliferation or involution, that grow possibly due to hemodynamic mechanisms (1,4,5). They are classified into low-flow, mixed and high-flow malformations (3,6). Venous malformations are common in low flow lesions and 60% of the cases occur in the craniofacial region (1). They usually are bluish soft nodules, formed by a network of thin-walled venous channels. History and physical examination is still the pillar for the diagnosis of vascular lesions; however, histology and immunohistochemistry can be helpful for classification. It is considered that GLUT-1 is negative in endothelial cells of vascular malformations, but positive in infantile hemangiomas (IH), helping for the correct diagnosis (4,5,7,8).

Vascular hemangiomas/malformations of the mouth are relatively frequent, but encapsulated vascular lesions in general are uncommon and, for the best of our knowledge, not yet reported in the oral cavity. We report a case of an encapsulated vascular malformation of the right buccal mucosa of an adult male patient.

## Case Report

**Figure 1:**
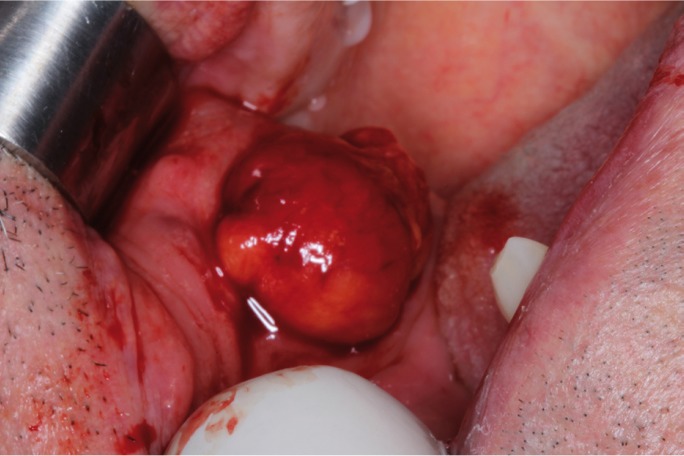
Clinical aspect during surgical excision of the encapsulated, well-defined and yellowish red nodule in the buccal mucosa, of approximately 20mm. The final diagnosis was of encapsulated vascular malformation.

A 69-year man was referred to our service with a swelling in the right cheek, of unknown time of evolution. Medical history was not contributory. Physical examination revealed a bluish soft asymptomatic nodule in the right buccal mucosa (Fig. 1).

**Figure 2:**
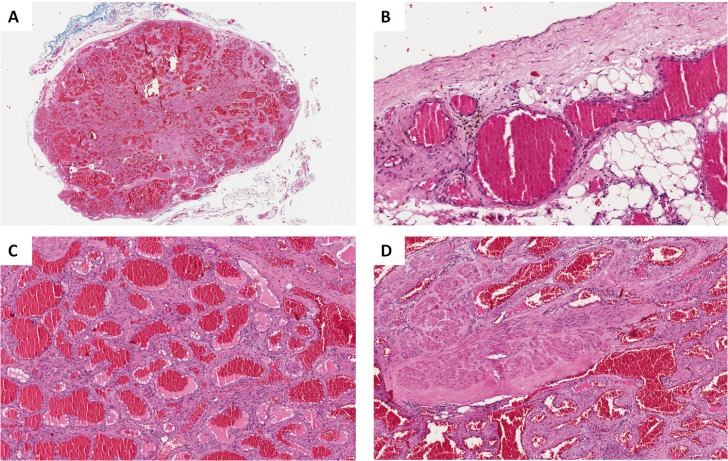
A) Low power magnification showing a well circumscribed and encapsulated lesion composed by a high number of plethoric vascular channels (H&E, OM, X5). B) Higher power view showing the fibrous capsule surrounding the lesion, and the presence of adipose tissue (H&E, OM, X100). C) Low power magnification showing the center of the lesion composed by ectatic blood vessels of variable size (H&E, OM, X100). D) Low power magnification showing bundles of smooth muscle in the stroma, unrelated to vessel walls (H&E, OM, X100).

An excisional biopsy was performed and microscopic examination revealed a well-encapsulated lesion rich in plethoric blood vessels lined by a single layer of flattened endothelial cells, presenting a lobular arrangement. The lesion was mainly composed by a venous component, and enlarged and tortuous vessels, with diameters ranging from 80 µm up to 1.5 mm. Some vessels showed a thin muscular wall, but more often large vessels presented thick walls. The stroma was collagenized and some areas displayed dense bundles of smooth muscle. Adipose tissue was also observed, mostly adjacent to the fibrous capsule (Fig. 2). Masson trichromic and picrosirius red, seen by polarized light, helped to highlight the evident capsule and the intervascular stroma.

**Figure 3:**
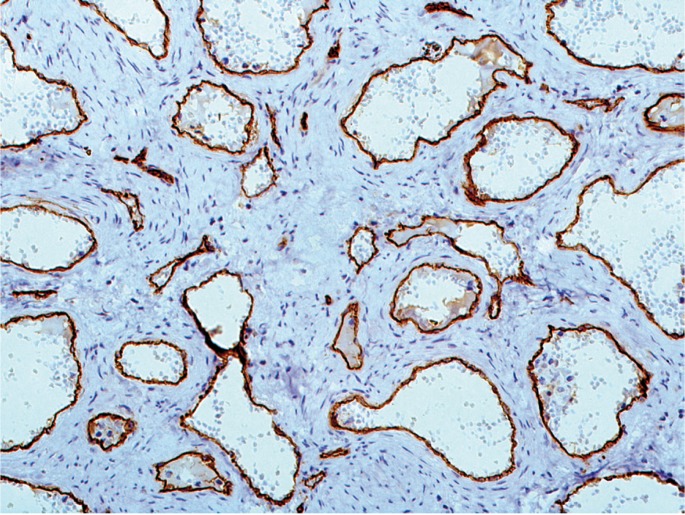
CD 34 highlighting the endothelial cells in blood vessels of various sizes. The stromal cells are negative (Immunohistochemistry, OM X200).

**Figure 4:**
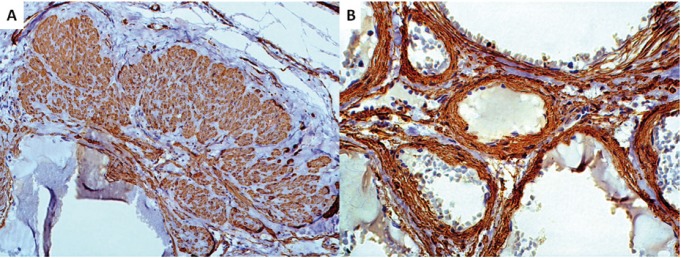
A) Smooth muscle bundles not directly associated with blood vessels, strongly positive for SMA, were found in some areas of the lesion (Immunohistochemistry, OM, X200). B) Vascular walls showing smooth muscle positive for SMA (Immunohistochemistry, OM, X400).

Antibodies used for immunohistochemistry are listed in  Table 1. CD-34 was positive for endothelial cells of all vessels (Fig. 3), while Ki-67 was less than 1%. Smooth muscle actin (SMA) and HHF-35 were positive for the muscular structures and myofiboblasts scattered among the collagen fibers (Fig. 4). Smooth muscle was observed either as a mural component and in areas adjcacent to the vascular structures. Positiveness for h-caldesmon, helped to better evaluate smooth muscle distribution around and between the vascular spaces, highlighting that thin and thick-walled vessels composed the lesion. GLUT-1 was negative in the endothelial cells and strongly positive in erythrocytes, used as internal control. After histopathological and immunohistochemical analysis the lesion was diagnosed as encapsulated vascular malformation.

**Table 1:**
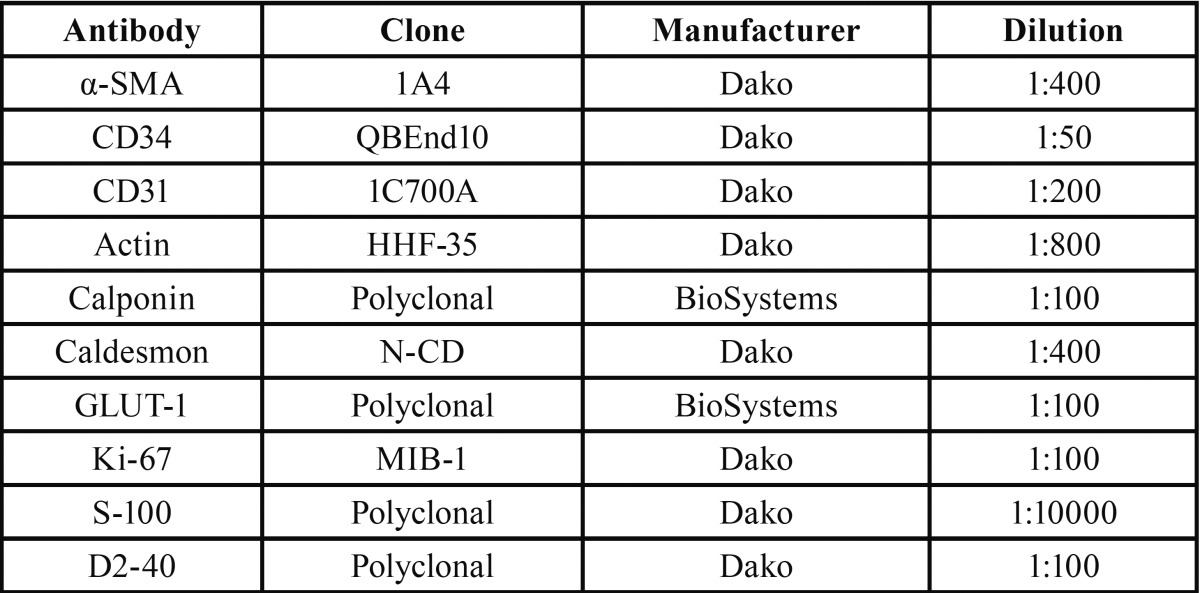
Antibodies used for immunohistochemistry in the case of encapsulated vascular malformation of the mouth.

## Discussion

Nomenclature of reactive/benign vascular lesions is not clear, but in general they can be considered either as hemangiomas or vascular malformations (1-3,9,10). In fact, it is common for pathologists to use the broad term hemangioma for most, if not all, benign vascular lesions formed by blood vessels, and eventually classify in capillary, cavernous or arteriovenous, according to the vascularization pattern (8). For an adequate diagnosis, clinical characteristics are of paramount relevance, but histological and immunohistochemical features can also be helpful. A correct classification is clinically relevant because biological behaviour and treatment can be different. Contrary to hemangiomas, vascular malformations are not considered as self-limiting, although disproportionate growth can occur, secondary to infection, trauma, hormonal influences, or progressive hemodynamic forces (8). According to the International Society for the Study of Vascular Anomalies (ISSVA), hemangiomas represent benign endothelial tumors characterized by rapid growth during infancy followed by a gradual involution. On the other hand, malformations are congenital, although not necessarily clinically apparent at birth, grow proportionally with the patient and do not have spontaneous regression (8,11). Despite the increasing general acceptance of the ISSVA classification, difficulties in diagnosis and treatment still persist (5,6).

Encapsulated vascular lesions (EVL) are uncommon and, for the best of our knowledge, this is an undescribed feature that vascular malformations can present in the oral cavity. According to Osaki *et al.* (2013), orbital EVL, affect predominantly adults, with a mean age of 48 years; with a slight predilection for men, while orbital hemangiomas appear shortly after birth, therefore patient’s age seems to be critical for diagnosis. Histopathological features of orbital EVL are similar to those found in the current case, such as a well-circumscribed and encapsulated lesion showing an evident fibrous capsule of variable thickness, rich in vascular channels, mostly plethoric of blood (3). Many lumens show plasma and compaction of erythrocytes, reflecting the stagnant circulation. Intervascular connective septa are slightly collagenized and vessels have a variably thickened muscular wall. Another finding is the presence of intraluminal thrombi at various stages of organization (3).

In the differential diagnosis of the present case, a few rare well-defined vascular intraoral lesions can be considered, as sinusoidal hemangioma and spindle cell hemangioma. The first was described as a well-circumscribed lesion, located in the submucosa, and composed of multiple lobules of greatly dilated, thin-walled, ramifying and anastomosing vascular channels, demonstrating a sinusoidal appearance. In deeper areas it is observed intercommunicating cavernous vessels filled with blood and lined by a single layer of flattened endothelial cells (12). However, a fibrous capsule was not described in this lesion and the presence of an elongated pseudopapillary growth observed at the superficial part of the sinusoidal hemangioma was absent in the current case. Spindle cell haemangioma was described as a unique vascular tumor with combined microscopic features of both cavernous hemangioma and Kaposi sarcoma (13). It is a submucosal tumor, well demarcated, but not encapsulated. It consists of an admixture of cavernous hemangioma-like elements and solid cellular areas, composed of bland spindle and epithelioid cells, some containing cytoplasmic vacuoles. Also it can be observed variably dilated and partially collapsed thin-walled vessels, lined by a monolayer of flat endothelial cells, with the lumen filled with blood and organizing thrombi (12-14). Although, this is a well-defined lesion, the fibrous capsule is absent and its biphasic pattern was not observed in the current case. Masson´s hemangioma/Tumor (intravascular papillary endothelial hyperplasia) is another differential diagnosis to be considered. It is relatively common in the mouth, but it is characteristically formed by endothelial proliferation, resulting in multiple papillary projections into a large vascular lumen, usually following traumatic thrombosis of a medium sized vessel, therefore quite different from the present case (15).

The fibrous capsule observed in the current case was the hallmark, with similar features as previously described in the orbital region and brain. Osaki *et al.* (2013) named the orbital lesion as Adult Encapsulated Venous Lesion (AEVL), presenting similar microscopic aspects (3). Therefore, we considered that the current lesion in the mouth also should be classified as an AEVL. In the brain, encapsulated hematoma is a well-defined entity that can result from vascular malformations or trauma and exceptionally it can evolve to an encapsulated hemangiomatous lesion, nevertheless it is considered distinct from AEVL (16). The presence of adipose tissue, albeit discreet, is another feature of the current lesion, but it was not possible to determine if it is a result of senescence, chronic injury or correspond to an hamartomatous growth (17).

Immunohistochemistry has been considered useful to differentiate hemangiomas and vascular malformations. Ki-67 is practically negative in malformations, but it can show a proliferative rate of up to 16% in hemangiomas (3). The proliferation index of the current lesion was very low, compatible with a malformation. CD31 and CD34 were positive for endothelial cells, while smooth muscle actin revealed a wide variability in the thickness of the myoid mural cells. The bundles of smooth muscle unrelated to vessel wall and scattered myofibroblasts, found in various areas of the present case, were important findings of the current lesion, that are also described in venous malformations (3). GLUT-1 is an erythrocyte-type glucose transporter protein expressed by endothelial cells at sites of blood-tissue barriers such as the central nervous system, placenta, retina, and peripheral nerves, but it is negative in the vasculature of the skin and soft tissues (4,7). GLUT-1 seems to be strongly and diffusely expressed in the endothelial cells at all stages of evolution and involution of hemangiomas. It has been considerated useful to differentiate hemangioma from vascular malformations, because it is not expressed in the endothelial cells of the latter (3,7-9,18). Endothelial cells of the present case were negative for GLUT-1, favoring the diagnosis of vascular malformation.

According to the clinical, histological and immunohistochemical features, the present case was diagnosed as an oral encapsulated venous malformation, similarly to the case reported in the orbit by Osaki *et al.* (2013) (3). Venous malformations are described as heterogeneous and complex lesions that can be superficial or deep, usually bluish and asymptomatic (19,20). It is possible that these lesions are congenital, becoming detectable in adults due to hemodynamic alterations, acquiring a greater size (3). In conclusion, encapsulated vascular malformations are uncommon, but can be found in the oral cavity.
